# ﻿Description of six new crab-spider species and first description of the male of *Phartaxizang* Liu & Yao, 2023 from Medog, Xizang, China (Araneae, Thomisidae)

**DOI:** 10.3897/zookeys.1217.127555

**Published:** 2024-11-01

**Authors:** Lu-Yu Wang, Yan-Nan Mu, Qian-Le Lu, Yong-Qiang Xu, Hai-Tao Bu, Feng Zhang, Zhi-Sheng Zhang

**Affiliations:** 1 Key Laboratory of Eco-environments in Three Gorges Reservoir Region (Ministry of Education), School of Life Sciences, Southwest University, Chongqing 400715, China Southwest University Chongqing China; 2 College of Life Sciences and Oceanography, Shenzhen University, Shenzhen 518000, China Shenzhen University Shenzhen China; 3 Institute of Plateau Biology of Xizang Autonomous Region, Lhasa 850001, Xizang Autonomous Region, China Institute of Plateau Biology of Xizang Autonomous Region Lhasa China; 4 Medog Biodiversity Observation and Research Station of Xizang Autonomous Region, Medog, China Medog Biodiversity Observation and Research Station of Xizang Autonomous Region Medog China; 5 Key Laboratory of Zoological Systematics and Application, College of Life Sciences, Hebei University, Baoding, Hebei 071002, China Hebei University Baoding China

**Keywords:** Description, morphology, new species, taxonomy

## Abstract

Seven species of the crab-spider family Thomisidae from Medog, Xizang, China are described here, including six new species: *Camaricusmedog* Wang, Lu & Zhang, **sp. nov.** (♂♀), *Monaesesxizang* Wang, Lu & Zhang, **sp. nov.** (♂♀), *Sinothomisusbeibeng* Wang, Lu & Zhang, **sp. nov.** (♂♀), *Sinothomisusdawai* Wang, Lu & Zhang, **sp. nov.** (♂♀), *Spilosynemamotuo* Wang, Lu & Zhang, **sp. nov.** (♂♀), and *Thomisusyarang* Wang, Lu & Zhang, **sp. nov.** (♂♀). The male of *Phartaxizang* Liu & Yao, 2023 is described here for the first time. Descriptions and photographs of all the species are provided.

## ﻿Introduction

Medog, a county seat of Nyingchi City, located downstream of the Yarlung Zangbo River, is a key transitional zone between the Eastern Himalayas and Hengduan Mountain region. The topography of Medog is complex and with a variety of vegetation types from tropical valley monsoon rain forest to cold ice sheet between the Yarlung Zangbo Grand Canyon to the summit of Nangabawa (Wang and Peng 2022). Medog is the most northerly tropical region in the Northern Hemisphere and is affected by a subtropical humid climate due to the warm and humid airflow from the Indian Ocean. It is the lowest area of the Tibetan Plateau, with the best hydrothermal conditions and the greatest precipitation (Li et al. 2024). The unique geographical environment and climate have contributed to the rich biodiversity of Medog. However, due to the remote location, complex environment, and inconvenient transportation, the research of spider diversity in Medog has not been achieved until recently with improvements in transportation. According to preliminary statistics, only 34 species have been recorded from Medog, most of them published since 2017 (Table 1).

**Table 1.** List of spider species recorded from Medog.

Family	Species	Distribution	References
Agelenidae	*Draconariusmedogensis*	Medog	Zhu et al. 2017
	*Draconariussubaspinatus*	Medog	
Anapidae	*Sinanapismedogensis*	Medog	Zhang and Lin 2018
Araneidae	*Araneusmotuoensis*	Medog	Yin et al. 1990
	*Argiopebeibeng*	Medog	Mi et al. 2024
	*Argiopecaesarea*	China (Xiang and Yunnan), India and Myanmar	
Clubionidae	*Clubionamedog*	Medog	Zhang et al. 2007
Corinnidae	*Apochinommamedog*	Medog	Zhang and Zhang 2023
Ctenidae	*Amauropelmamedogensis*	Medog	Wang et al. 2024a
	*Anahitamedog*	Medog, Chayu of Xizang	Chu et al. 2022
	*Bowierotundus*	Medog	Wang et al. 2024a
Halonoproctidae	*Conothelemedoga*	Medog	Zhang and Yu 2021
Lycosidae	*Serratacosamedogensis*	Medog	Wang et al. 2021
	*Zantheresgracillimus*	Medog; Bhamo of Myanmar	Wang and Zhang 2020
Oonopidae	*Ischnothyreusmetok*	Medog	Tong et al. 2023
	*Paramolotrametok*	Medog	Cheng et al. 2021
Pholcidae	*Belisanamedog*	Medog	Zhu et al. 2020
	*Pholcusmedog*	Medog	Zhang et al. 2006
Pisauridae	*Hygropodamedogensis*	Medog	Lu et al. 2023
Psilodercidae	*Leclerceraaniensis*	Medog	Chang and Li 2020
	*Leclerceraduandai*	Medog	
	*Leclercerarenqinensis*	Medog	
	*Merizoceranyingchi*	Medog	Chang et al. 2020
Salticidae	*Chrysillayarlungzangbo*	Medog	Yang and Zhang 2024
	*Hyllusqishuoi*	Medog	Xiong et al. 2017
	*Synagelidesmedog*	Medog	Wang et al. 2024
Sparassidae	*Pseudopodaconica*	Medog	Zhang et al. 2023
	*Pseudopodamedogensis*	Medog	Jiang et al. 2018
	*Pseudopodashuo*	Medog	
	*Pseudopodazhangi*	Medog	Fu and Zhu 2008
Theraphosidae	*Chilobrachysjinchengi*	Medog	Lin et al. 2022
Thomisidae	*Phartaxizang*	Medog	Li et al. 2023
Zodariidae	*Asceuadawai*	Medog	Wang et al. 2024b
	*Mallinella mеdog*	Medog	

Our team has been investigating the spider diversity of Medog since 2018, and we have found more than 300 species, including many undescribed species. Here, seven crab-spider species are described, including six new species and the male of *Phartaxizang* new to science.

## ﻿Materials and methods

All specimens are preserved in 75% ethanol and were examined, illustrated, photographed, and measured using a Leica M205A stereomicroscope equipped with a Leica DFC450 Camera and LAS software (v. 4.6). Male pedipalps and epigynes were examined and illustrated after dissection. Epigynes were cleared by immersing them in a pancreatin solution (Álvarez-Padilla and Hormiga 2007). Eye sizes were measured as the maximum dorsal diameter. Leg measurements are shown as: total length (femur, patella and tibia, metatarsus, tarsus). All measurements are in millimetres. All specimens, including the holotypes examined here, are deposited in the Collection of Spiders, School of Life Sciences, Southwest University, Chongqing, China (**SWUC**).

Terminology follows Tang and Li (2010). Abbreviations used in the text: **ALE**–anterior lateral eye; **AME**–anterior median eye; **MOA**–median ocular area; **PLE**–posterior lateral eye; **PME**–posterior median eye.

## ﻿Taxonomy

**Family Thomisidae Sundevall, 1833** (蟹蛛科)

**Genus *Camaricus* Thorell, 1887** (顶蟹蛛属)

### ﻿ Camaricus medog

Taxon classificationAnimaliaAraneaeThomisidae

Wang, Lu & Zhang sp. nov.

A40D835C-D548-587F-9D7A-771A4BBFF746

https://zoobank.org/D88E4BFE-1E8B-4050-B5CE-29730C9E751B

**Figure 1.**
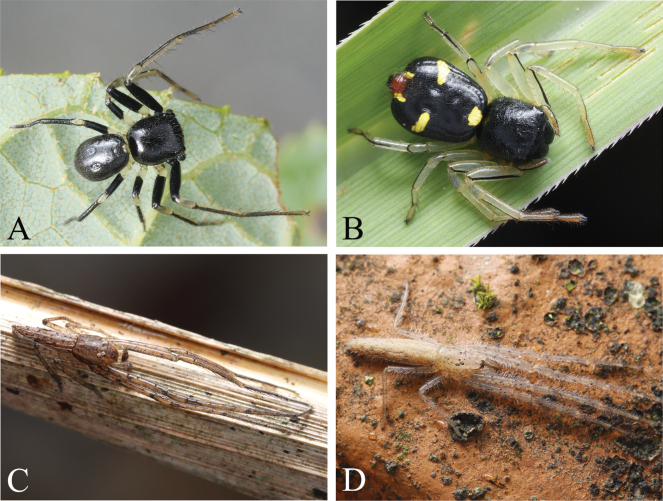
Living photos of crab spiders **A, B***Camaricusmedog* Wang, Lu & Zhang, sp. nov. **A** male holotype **B** female paratype **C, D***Monaesesxizang* Wang, Lu & Zhang, sp. nov. **C** male holotype, **D** female paratype. Photographed by Qian-Le Lu.

Figs 1A, B , 3A, B , 4 , 11

#### Type material.

***Holotype*** • ♂ (SWUC-T-THO-01-01), China, Xizang, Medog County, Mirage observation deck, 29°20'36"N, 95°20'43"E, elev. 1297 m, 8 July 2023, Z.S. Zhang, L.Y. Wang, Q.L. Lu and X.L. Chen leg. ***Paratypes***: • 1 ♂ 4 ♀ (SWUC-T-THO-01-02~06), same data as for holotype.

#### Etymology.

The specific name is derived from the type locality; it is a noun in apposition.

#### Diagnosis.

The new species resembles *C.formosus* Thorell, 1887 (Song and Zhu 1997: 173, fig. 122A–D) in having the embolus origin in the same position, but *C.medog* differs from the latter by the short and blunt ventral tibial apophysis end (vs long and sharp in *C.formosus*), a bifurcated retrolateral tibial apophysis (vs single in *C.formosus*), a broad embolus (vs slender in *C.formosus*), and a distinct copulatory atrium (vs indistinct in *C.formosus*) (Fig. 4).

#### Description.

**Male** holotype (SWUC-T-THO-01-01, Figs 1A, 3A) total length 4.50. Prosoma 2.40 long, 2.09 wide; opisthosoma 2.03 long, 1.69 wide. Carapace black. Eye sizes and interdistances: AME 0.12, ALE 0.16, PME 0.05, PLE 0.12; AME–AME 0.39, AME–ALE 0.34, PME–PME 0.68, PME–PLE 0.51, ALE–PLE 0.31. MOA 0.33 long, anterior width 0.66, posterior width 0.82. Clypeus height 0.21. Chelicerae black, with 3 promarginal and 3 retromarginal teeth. Labium and endites brown, longer than wide. Sternum brown and scutellate. Leg measurements: I 7.65 (2.22, 2.85, 1.55, 1.03); II 7.58 (2.21, 2.83, 1.48, 1.06); III 4.39 (1.45, 1.60, 0.69, 0.65); IV 4.37 (1.44, 1.62, 0.74, 0.57). Leg formula: 1234. Opisthosoma oval, black, with a white spot at mid-anterior part. Spinnerets black.

***Palp*** (Fig. 4A, B). Tibial as 1/2 of length cymbium, ventral tibial apophysis somewhat pediform, retrolateral tibial apophysis bifurcated: ventral arm thumb shaped, retrolateral arm hook-like in retrolateral view. Embolus originating at approximately 9-o’clock position, broad, curved along with bulb.

**Female** paratype (SWUC-T-THO-01-02, Fig. 3B) total length 6.60. Prosoma 3.04 long, 2.63 wide; opisthosoma 3.70 long, 3.22 wide. Eye sizes and interdistances: AME 0.11, ALE 0.21, PME 0.10, PLE, 0.19; AME–AME 0.49, AME–ALE 0.46, PME–PME 0.84, PME–PLE 0.60, ALE–PLE 0.32. MOA 0.45 long, anterior width 0.80, posterior width 1.04. Clypeus height 0.27. Leg measurements: I 7.07 (2.10, 2.55, 1.40, 1.02); II 7.16 (2.13, 2.70, 1.37, 0.96); III 4.72 (1.59, 1.77, 0.75, 0.61); IV 5.38 (1.74, 2.13, 0.87, 0.64). Leg formula: 2143. Opisthosoma oval, black, with 5 white (yellow when live) and one brown spots. Spinnerets black.

***Epigyne*** (Fig. 4C, D). Epigynal plate longer than wide. Copulatory opening located anteriorly. Copulatory ducts transparent and C-like. Spermathecae stomach shaped. Fertilization ducts crescent-shaped.

#### Variation.

Males (*n = 2*) total length 4.50–4.92; females (*n = 4*) total length 6.60–8.00.

#### Distribution.

Known only from the type locality, Medog, Xizang, China (Fig. 11).

##### ﻿Genus *Monaeses* Thorell, 1869 (莫蟹蛛属)

### ﻿ Monaeses xizang

Taxon classificationAnimaliaAraneaeThomisidae

Wang, Lu & Zhang sp. nov.

DBC2B6A7-F40B-5617-BF51-22735FCC7EF3

https://zoobank.org/6E866803-36E5-4324-8E7A-F8F686965881

Figs 1C, D , 3M, N , 5 , 11

#### Type material.

***Holotype*** • ♂ (SWUC-T-THO-02-01), China, Xizang, Medog County, Mirage Observation Deck, 29°20'36"N, 95°20'43"E, elev. 1297 m, 8 July 2023, Z.S. Zhang, L.Y. Wang, Q.L. Lu and X.L. Chen leg. ***Paratype***: • 1 ♀ (SWUC-T-THO-02-02), same data as for holotype.

#### Etymology.

The specific name is derived from the type locality; it is a noun in apposition.

#### Diagnosis.

The new species resembles *M.aciculus* (Simon, 1903) (Song and Zhu 1997: 59, fig. 35A–H) in having the ventral tibial apophysis of the same shape and spermathecae compressed, but the new species differs from *M.aciculus* in having the ventral tibial apophysis shorter than retrolateral tibial apophysis (vs as long as retrolateral tibial apophysis in *M.aciculus*), a short embolus originating at approximately 11-o’clock (vs long and originating at approximately 8-o’clock in *M.aciculus*), and comma-like copulatory ducts (vs U-shaped in *M.aciculus*) (Fig. 5).

#### Description.

**Male** holotype (SWUC-T-THO-02-01, Figs 1C, 3M) total length 7.31. Prosoma 2.24 long, 1.82 wide; opisthosoma 5.33 long, 1.24 wide. Carapace yellow brown. Eye sizes and interdistances: AME 0.06, ALE 0.14, PME 0.09, PLE 0.18; AME–AME 0.17, AME–ALE 0.19, PME–PME 0.34, PME–PLE 0.29, ALE–PLE 0.31. MOA 0.40 long, anterior width 0.30, posterior width 0.51. Clypeus height 0.45. Chelicerae yellow brown. Labium and endites yellow brown, longer than wide. Sternum yellow-brown and scutellate, with brown hairs. Leg measurements: I 18.79 (5.43, 6.98, 4.57, 1.81); II 16.96 (5.06, 6.25, 3.94, 1.71); III 6.61 (2.10, 2.57, 1.09, 0.85); IV 7.81 (3.28, 2.52, 1.08, 0.93). Leg formula: 1243. Opisthosoma columnar, yellow brown. Spinnerets yellow-brown.

***Palp*** (Fig. 5A, B). Tibia ½ length of cymbium. Ventral tibial apophysis columnar, with a curved end. Retrolateral tibial apophysis sclerous, somewhat triangular, with a sharp end. Embolus originating at approximately 11-o’clock position, slender, curved along with bulb, tip staying in cymbial furrow.

**Female** paratype (SWUC-T-THO-02-02, Figs 1D, 3N) total length 10.70. Prosoma 3.23 long, 2.35 wide; opisthosoma 7.28 long, 1.88 wide. Eye sizes and interdistances: AME 0.07, ALE 0.22, PME 0.11, PLE, 0.19; AME–AME 0.32, AME–ALE 0.27, PME–PME 0.52, PME–PLE 041, ALE–PLE 0.39. MOA 0.49 long, anterior width 0.46, posterior width 0.74. Clypeus height 0.58. Leg measurements: I 16.12 (5.05, 6.17, 3.34, 1.56); II 14.41 (4.53, 5.52, 2.88, 1.48); III 14.30 (4.39, 5.53, 2.98, 1.40); IV 8.37 (3.57, 2.81, 1.11, 0.88). Leg formula: 1234.

***Epigyne*** (Fig. 5C, D). Epigynal plate almost rounded. Copulatory openings slit-like, distant from each other. Copulatory ducts comma-like. Spermathecae folded. Fertilization ducts crescent-shaped.

#### Distribution.

Known only from the type locality, Medog, Xizang, China (Fig. 11).

##### ﻿Genus *Pharta* Thorell, 1891 (范蟹蛛属)

### ﻿ Pharta xizang

Taxon classificationAnimaliaAraneaeThomisidae

Liu & Yao, 2023

5EF8BCFC-9C1A-5816-B1A7-130FD948E488

**Figure 2.**
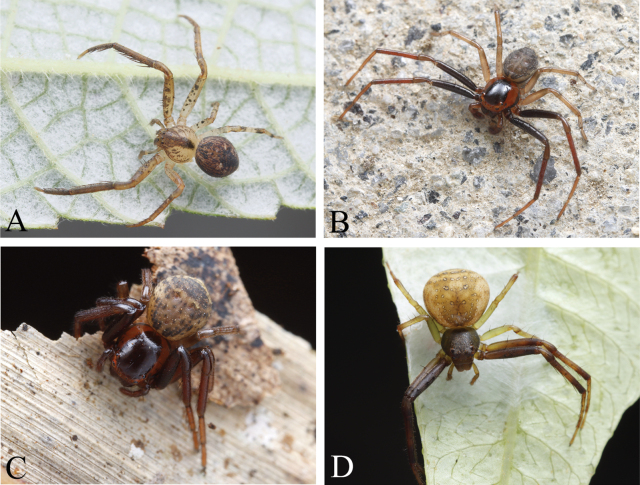
Living photos of crab spiders **A***Phartaxizang* Liu & Yao, 2023, female **B, C***Sinothomisusdawai* Wang, Lu & Zhang, sp. nov. **B** male holotype **C** female **D***Spilosynemaxizang* Wang, Lu & Zhang, sp. nov., female paratype. Photographed by Qian-Le Lu.

**Figure 3.**
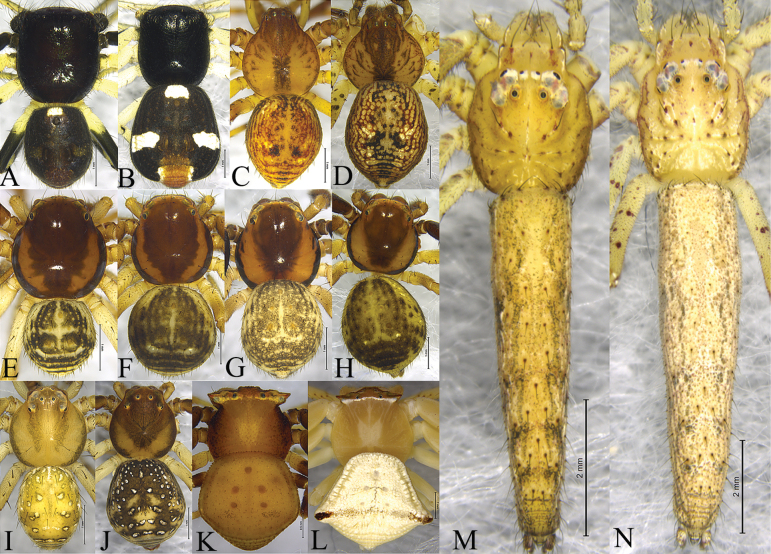
Habitus, dorsal view **A, B***Camaricusmedog* Wang, Lu & Zhang, sp. nov. (**A** male holotype **B** female paratype) **C, D***Phartaxizang* Liu & Yao, 2023 (**C** male **D** female) **E, F***Sinothomisusbeibeng* Wang, Lu & Zhang, sp. nov. (**E** male holotype **F** female paratype) **G, H***Sinothomisusdawai* Wang, Lu & Zhang, sp. nov. (**G** male holotype **H** female paratype) **I, J***Spilosynemaxizang* Wang, Lu & Zhang, sp. nov. (**I** male holotype **J** female paratype) **K, L***Thomisusyarang* Wang, Lu & Zhang, sp. nov. (**K** male holotype, **L** female paratype) **M, N***Monaesesxizang* Wang, Lu & Zhang, sp. nov. (**M** male holotype **N** female paratype).

Figs 2A , 3C, D , 6 , 11

Pharta xizang Liu & Yao in Li et al., 2023: 176, fig. 4A–G (♀).

#### Material examined.

**China, Xizang, Medog County**: • 2 ♂, Medog Town, 29°19.470'N, 95°19.618'E, elev. 1116 m, 27 June 2018, L.Y. Wang, Z.S. Wu and Y.N. Mu leg; • 1 ♀, Beibeng Township, Damu, 29°14'51"N, 95°11'1"E, elev. 924 m, 28 June 2018, L.Y. Wang, Y.N. Mu and Z.S. Wu leg; • 1 ♀, Medog Town, Yarang Village, 29°17.758'N, 95°16.827'E, elev. 761 m, 22 May 2019, L.Y. Wang, T. Yuan, P. Liu and H. Wang leg; • 1 ♀, Medog Town, 23 May 2019, L.Y. Wang, T. Yuan, P. Liu and H. Wang leg; • 2 ♂ 4 ♀, Beibeng Township, Badeng Village, 29°16'28"N, 95°10'7"E, elev. 851 m, 7 July 2023, Z.S. Zhang, L.Y. Wang, Q.L. Lu and X.L. Chen leg; • 2 ♂, Mirage Observation Deck, 29°20'36"N, 95°20'43"E, elev. 1297 m, 8 July 2023, Z.S. Zhang, L.Y. Wang, Q.L. Lu and X.L. Chen leg.

#### Diagnosis.

This species resembles *P.tangi* Wang, Mi & Peng, 2016 (Wang et al. 2016: 130, figs 1A–G, 2A–D) in having similar shaped spermathecae, but differs it from the latter by the knife-like ventral tibial apophysis (vs rhabditiform and slightly curved in *P.tangi*), the multiple spines at the base of ventral tibial apophysis (vs spine absent in *P.tangi*), the folded posterior half part of conductor (vs knife-shaped in *P.tangi*), and the kidney-shaped spermathecae (vs long oval in *P.tangi*) (Fig. 6). This new species also resembles *P.gongshan* (Yang, Zhu & Song, 2006) (Benjamin 2011: 80, figs 47A, B, D–F, 49A–F, 51A–D) in having a similar shaped bulb, but it can be distinguished by the bifurcated tip of the conductor (vs not bifurcated in *P.gongshan*) and the small copulatory openings located towards the posterior (vs large copulatory openings towards the lateral).

#### Description.

**Male** (Fig. 3C) total length 5.89. Prosoma 2.74 long, 2.40 wide; Opisthosoma 3.08 long, 2.43 wide. Carapace yellow-brown, with a deep-brown spot. Eye sizes and interdistances: AME 0.07, ALE 0.20, PME 0.16, PLE 0.18; AME–AME 0.12, AME–ALE 0.07, PME–PME 0.14, PME–PLE 0.16, ALE–PLE 0.17. MOA 0.42 long, anterior width 0.26, posterior width 0.45. Clypeus height 0.15. Chelicerae brown, with 3 promarginal and 3 retromarginal teeth. Labium and endites yellow-brown, longer than wide. Sternum yellow-brown and scutellate, with brown hairs. Leg measurements: I 11.47 (3.39, 4.34, 2.51, 1.23); II 10.92 (3.24, 4.14, 2.28, 1.26); III 6.16 (1.97, 2.34, 1.13, 0.72); IV 7.25 (2.28, 2.61, 1.58, 0.78). Leg formula: 1243. Opisthosoma oval, yellow, with a deep-red spot. Spinnerets yellow-brown.

**Figure 4.**
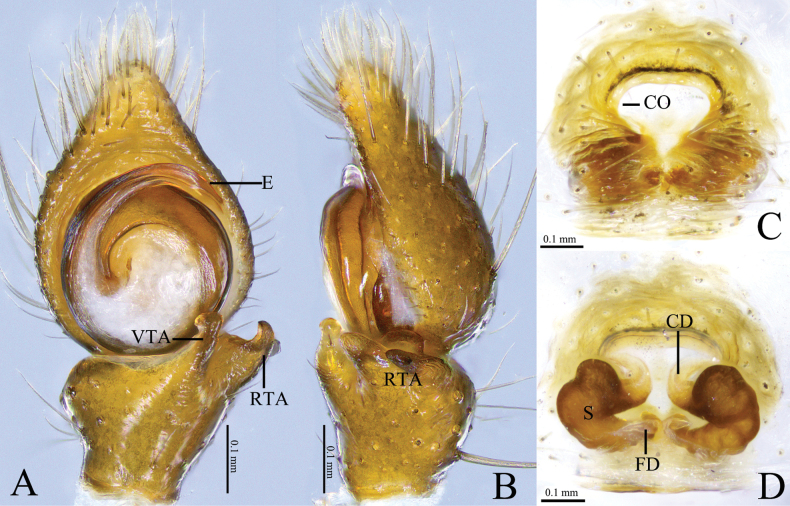
*Camaricusmedog* Wang, Lu & Zhang, sp. nov. **A, B** holotype male **C, D** paratype female **A** male left palp, ventral view **B** same, retrolateral view. **C** epigyne, ventral view **D** same, dorsal view. Abbreviations: CD = copulatory duct; CO = copulatory opening; E = embolus; FD = fertilization duct; RTA = retrolateral tibial apophysis; VTA = ventral tibial apophysis; S = spermathecal.

**Figure 5.**
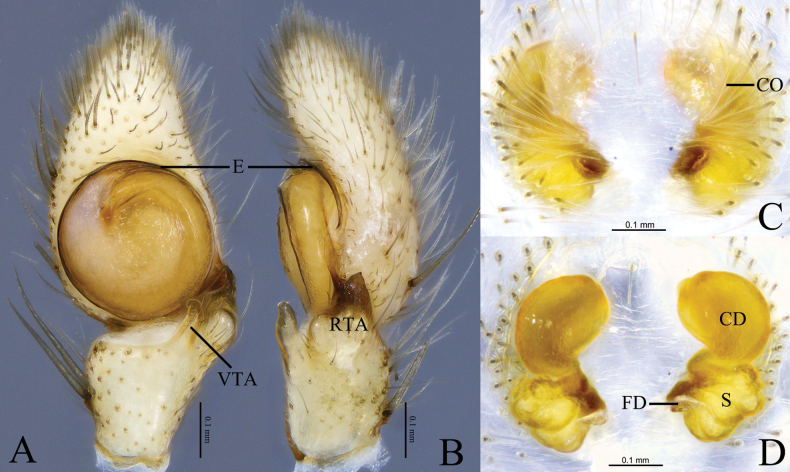
*Monaesesxizang* Wang, Lu & Zhang, sp. nov. **A, B** holotype male **C, D** paratype female **A** male left palp, ventral view **B** same, retrolateral view **C** epigyne, ventral view **D** same, dorsal view. Abbreviations: CD = copulatory duct; CO = copulatory opening; E = embolus; FD = fertilization duct; RTA = retrolateral tibial apophysis; VTA = ventral tibial apophysis; S = spermathecal.

**Figure 6.**
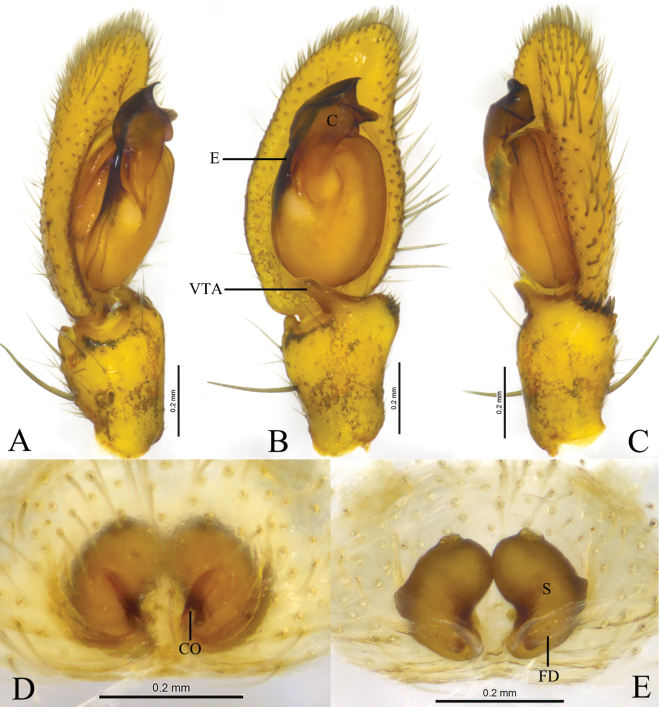
*Phartaxizang* Liu & Yao, 2023 **A** male left palp, prolateral view **B** same, ventral view **C** same, retrolateral view **D** epigyne, ventral view **E** same, dorsal view. Abbreviations: CD = copulatory duct; C = conductor; CO = copulatory opening; E = embolus; FD = fertilization duct; VTA = ventral tibial apophysis; S = spermathecal.

***Palp*** (Fig. 6A–C). Tibia longer than wide. Ventral tibial apophysis knife-like, three times longer than wide, base of ventral tibial apophysis bulging, with multiple spines. Conductor originating from centre of bulb, base wider than embolus, anterior part folded as a furrow. Embolus strong, originating at approximately 9-o’clock position, with its tip staying in conductor furrow.

**Female** (Fig. 3D) total length 7.20. Prosoma 3.04 long, 2.52 wide; opisthosoma 3.82 long, 3.47 wide. Eye sizes and interdistances: AME 0.06, ALE 0.19, PME 0.16, PLE, 0.19; AME–AME 0.14, AME–ALE 0.10, PME–PME 0.14, PME–PLE 0.19, ALE–PLE 0.19. MOA 0.45 long, anterior width 0.27, posterior width 0.48. Clypeus height 0.17. Leg measurements: I 11.07 (3.28, 4.61, 2.16, 1.02); II 10.28 (3.09, 4.21, 1.92, 1.06); III 5.90 (1.88, 2.37, 0.99, 0.66); IV 7.24 (2.30, 2.65, 1.55, 0.74). Leg formula: 1243.

***Epigyne*** (Fig. 6D, E). Epigynal plate almost rounded. Copulatory openings small, rounded, far away from each other. Copulatory ducts short. Spermathecae kidney-shaped. Fertilization ducts crescent-shaped.

#### Distribution.

China (Xizang, Medog) (Fig. 11).

##### ﻿Genus *Sinothomisus* Tang, Yin, Griswold & Peng, 2006 (华蟹蛛属)

### ﻿ Sinothomisus beibeng

Taxon classificationAnimaliaAraneaeThomisidae

Wang, Lu & Zhang sp. nov.

D79D8D05-68D2-522D-9DB2-4DBC0F22BB15

https://zoobank.org/99A666E2-A89A-44A9-B224-ECA5C062D6CD

Figs 3E, F , 7 , 11

#### Type material.

***Holotype*** • ♂ (SWUC-T-THO-03-01), China, Xizang, Medog County, Beibeng Township, Damu, 29°14'51"N, 95°11'1"E, elev. 924 m, 28 June 2018, L.Y. Wang, Y.N. Mu and Z.S. Wu leg. ***Paratype***: • 1 ♀ (SWUC-T-THO-03-02), same data as for holotype.

#### Etymology.

The specific name is derived from the type locality; it is a noun in apposition.

#### Diagnosis.

**Figure 7.**
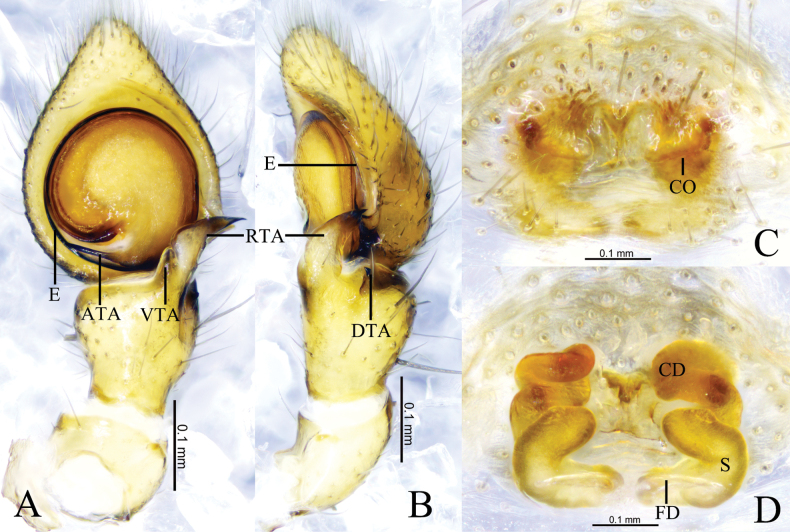
*Sinothomisusbeibeng* Wang, Lu & Zhang, sp. nov. **A, B** holotype male **C, D** paratype female **A** male left palp, ventral view **B** same, retrolateral view **C** epigyne, ventral view **D** same, dorsal view. Abbreviations: CD = copulatory duct; CO = copulatory opening; DTA = dorsal tibial apophysis; E = embolus; FD = fertilization duct; RTA = retrolateral tibial apophysis; VTA = ventral tibial apophysis; S = spermathecal.

The new species resembles *S.liae* Tang, Yin, Griswold & Peng, 2006 (Tang et al. 2006: 65, figs 1–13) in having a similar shaped retrolateral tibial apophysis, but it differs from the latter by the retrolateral tibial apophysis which is shorter than the bulb (vs equal in length to the bulb in *S.liae*), the long, thin apical tegular apophysis (vs short and wide in *S.liae*), and the C-like spermathecae (vs caterpillar-like in *S.liae*) (Fig. 7). This new species also resembles *S.dawai* Wang, Lu & Zhang, sp. nov.; see diagnosis for *S.dawai*.

#### Description.

**Male** holotype (SWUC-T-THO-03-01, Fig. 3E) total length 4.03. Prosoma 2.19 long, 2.12 wide; opisthosoma 1.96 long, 1.56 wide. Carapace border black, with a large, deep-brown spot in the middle. Eye sizes and interdistances: AME 0.08, ALE 0.17, PME 0.02, PLE 0.16; AME–AME 0.22, AME–ALE 0.23, PME–PME 0.26, PME–PLE 0.47, ALE–PLE 0.19. MOA 0.22 long, anterior width 0.37, posterior width 0.30. Clypeus height 0.22. Chelicerae brown. Labium and endites brown, longer than wide. Sternum brown and scutellate, with brown hairs. Leg measurements: I 8.09 (2.39, 2.89, 1.65, 1.16); II 8.36 (2.46, 3.07, 1.64, 1.19); III 5.27 (1.70, 1.96, 0.84, 0.77); IV 5.25 (1.71, 1.90, 0.94, 0.70). Leg formula: 2134. Opisthosoma oval, yellow, with a black spot. Spinnerets brown.

***Palp*** (Fig. 7A, B). Tibia longer than wide. Ventral tibial apophysis small, thumb-shaped, near retrolateral tibial apophysis. Retrolateral tibial apophysis four times longer than ventral tibial apophysis, jellyfish-shaped in retrolateral view. Dorsal tibial apophysis small, conical. Bulb flat, apical tegular apophysis thin and long. Embolus filiform, originating at approximately 6-o’clock position, curved along with bulb, tip staying in cymbial furrow.

**Female** paratype (SWUC-T-THO-03-02, Fig. 3F) total length 4.86. Prosoma 2.37 long, 2.17 wide; opisthosoma 2.48 long, 2.32 wide. Eye sizes and interdistances: AME 0.08, ALE 0.19, PME 0.02, PLE 0.16; AME–AME 0.23, AME–ALE 0.26, PME–PME 0.26, PME–PLE 0.52, ALE–PLE 0.23. MOA 0.26 long, anterior width 0.40, posterior width 0.32. Clypeus height 0.25. Leg measurements: I 6.90 (2.13, 2.60, 1.26, 0.91); II 7.16 (2.24, 2.62, 1.30, 1.00); III 4.85 (1.56, 1.87, 0.70, 0.72); IV 4.81 (1.60, 1.73, 0.81, 0.67). Leg formula: 2134.

***Epigyne*** (Fig. 7C, D). Epigynal plate as long as wide. Copulatory openings slit-like, distant from each other. Copulatory ducts S-like. Spermathecae C-shaped. Fertilization ducts crescent-shaped.

#### Distribution.

Known only from the type locality, Medog, Xizang, China (Fig. 11).

### ﻿ Sinothomisus dawai

Taxon classificationAnimaliaAraneaeThomisidae

Wang, Lu & Zhang sp. nov.

315BF461-E148-5A5D-BBA4-ADEEC77BD414

https://zoobank.org/36CEFA28-BE47-4CBD-8D15-BD72D8EAC7D9

**Figure 8.**
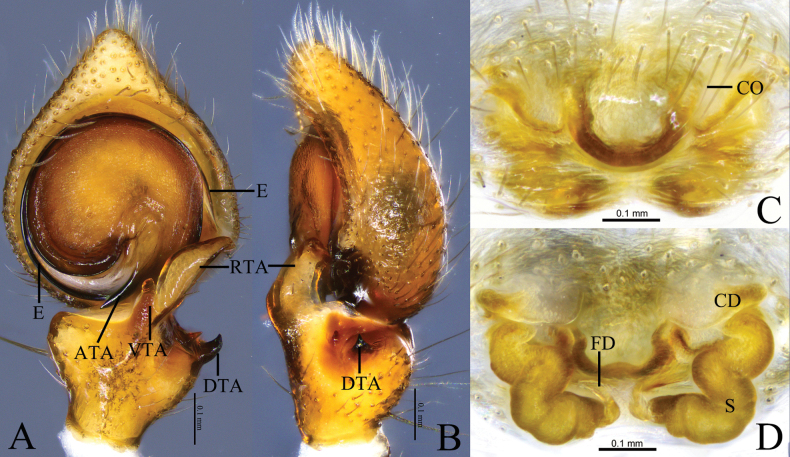
*Sinothomisusdawai* Wang, Lu & Zhang, sp. nov. **A, B** holotype male **C, D** paratype female **A** male left palp, ventral view **B** same, retrolateral view **C** epigyne, ventral view **D** same, dorsal view. Abbreviations: CD = copulatory duct; CO = copulatory opening; DTA = dorsal tibial apophysis; E = embolus; FD = fertilization duct; RTA = retrolateral tibial apophysis; VTA = ventral tibial apophysis; S = spermathecal.

Figs 2B, C , 3G, H , 8 , 11

#### Type material.

***Holotype*** • ♂ (SWUC-T-THO-04-01), China, Xizang, Medog County, Mirage Observation Deck, 29°20'36"N, 95°20'43"E, elev. 1297 m, 8 July 2023, Z.S. Zhang, L.Y. Wang, Q.L. Lu and X.L. Chen leg. ***Paratypes***: • 1 ♂ (SWUC-T-THO-04-02), same data as for holotype; • 1 ♀ (SWUC-T-THO-04-03), Beibeng Township, Damu, 29°14'51"N, 95°11'1"E, elev. 924 m, 28 June 2018, L.Y. Wang, Y.N. Mu and Z.S. Wu leg.

#### Etymology.

The specific name is a patronym in honor of Mr. Dawa from the Tibet Plateau Institute of Biology in Lhasa, Xizang.

#### Diagnosis.

The new species resembles *S.beibeng* Wang, Lu & Zhang, sp. nov. (Fig. 7) in having same shaped bulb and same position of embolus origin, but differs from the latter by the long and large retrolateral tibial apophysis (vs short and C-like in *S.beibeng*), the small spine between the retrolateral tibial apophysis and the dorsal tibial apophysis (vs absent in *S.beibeng*), the fingernail-like protuberance at epigynal plate (vs absent in *S.beibeng*) (Fig. 8).

#### Description.

**Male** holotype (SWUC-T-THO-04-01, Figs 2B, 3G) total length 4.21. Prosoma 2.19 long, 2.04 wide; Opisthosoma 2.34 long, 1.92 wide. Carapace border black, with a large deep brown spot in the middle. Eye sizes and interdistances: AME 0.07, ALE 0.18, PME 0.01, PLE 0.16; AME–AME 0.24, AME–ALE 0.21, PME–PME 0.24, PME–PLE 0.45, ALE–PLE 0.21. MOA 0.24 long, anterior width 0.37, posterior width 0.29. Clypeus height 0.18. Chelicerae brown. Labium and endites brown, longer than wide. Sternum brown and scutellate, with brown hairs. Leg measurements: I 8.40 (2.54, 3.04, 1.58, 1.24); II 8.52 (2.62, 3.07, 1.63, 1.20); III 5.42 (1.81, 1.98, 0.91, 0.72); IV 5.34 (1.70, 1.99, 0.95, 0.70). Leg formula: 2134. Opisthosoma oval, yellow, with black spot. Spinnerets brown.

***Palp*** (Fig. 8A, B). Tibia as long as wide. Ventral tibial apophysis small, thumb shaped, closing to retrolateral tibial apophysis. Retrolateral tibial apophysis middle part bulging, with a hook end. Dorsal tibial apophysis small, cone-shaped. A spine-like tubercle between retrolateral tibial apophysis and dorsal tibial apophysis. Bulb flat, apical tegular apophysis nail-shaped. Embolus filiform, originating at approximately 6-o’clock position, curved along with bulb, tip staying in cymbial furrow.

**Female** paratype (SWUC-T-THO-04-03, Fig. 3H) total length 5.59. Prosoma 2.28 long, 2.25 wide; opisthosoma 3.10 long, 2.61 wide. Eye sizes and interdistances: AME 0.08, ALE 0.18, PME 0.02, PLE 0.15; AME–AME 0.28, AME–ALE 0.25, PME–PME 0.25, PME–PLE 0.54, ALE–PLE 0.25. MOA 0.26 long, anterior width 0.46, posterior width 0.33. Clypeus height 0.22. Leg measurements: I 7.00 (2.19, 2.56, 1.30, 0.95); II 7.35 (2.30, 2.79, 1.28, 0.98); III 5.05 (1.65, 1.90, 0.76, 0.74); IV 4.84 (1.44, 1.86, 0.84, 0.70). Leg formula: 2134.

***Epigyne*** (Fig. 8C, D). Epigynal plate wider than long, with a fingernail-like protuberance at middle part. Copulatory openings slit like, far away from each other. Copulatory ducts S-like. Spermathecae C-shaped. Fertilization ducts crescent.

#### Variation.

Males (*n = 2*) total length 4.21–5.17.

#### Distribution.

China (Xizang, Medog) (Fig. 11).

##### ﻿Genus *Spilosynema* Tang & Li, 2010 (花斑蛛属)

### ﻿ Spilosynema motuo

Taxon classificationAnimaliaAraneaeThomisidae

Wang, Lu & Zhang sp. nov.

AE55BBBF-317B-553C-9E4E-7579AAA8311B

https://zoobank.org/C8CEDAA7-E58F-43AF-B415-3B0C06A84AE4

**Figure 9.**
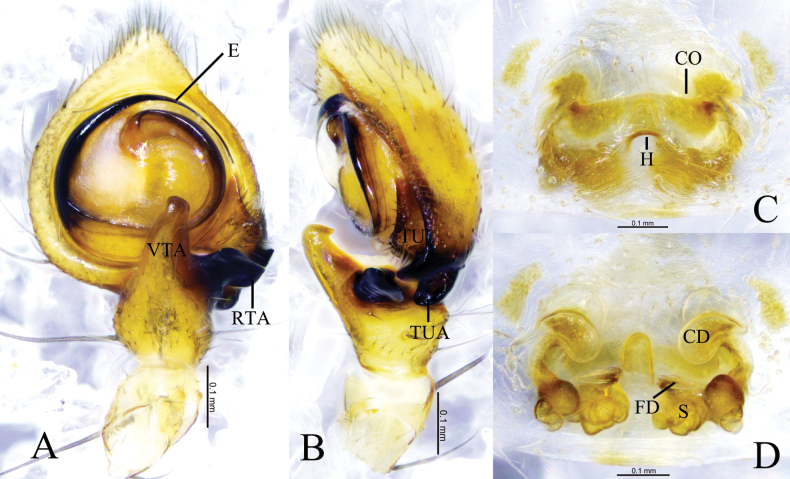
*Spilosynemamotuo* Wang, Lu & Zhang, sp. nov. **A, B** holotype male **C, D** paratype female **A** male left palp, ventral view **B** same, retrolateral view **C** epigyne, ventral view **D** same, dorsal view. Abbreviations: CD = copulatory duct; CO = copulatory opening; E = embolus; FD = fertilization duct; H = hood; RTA = retrolateral tibial apophysis; Tu = tutaculum; TUA = tutacular apophysis; VTA = ventral tibial apophysis; S = spermathecal.

Figs 2D , 3I–J , 9 , 11

#### Type material.

***Holotype*** • ♂ (SWUC-T-THO-05-01), China, Xizang, Medog County, Beibeng Township, 29°14.87'N, 95°11.02'E, elev. 924 m, 19 December 2023, Z.S. Zhang, L.Y. Wang, Q.L. Lu and Y.N. Mu leg. ***Paratypes***: • 1 ♀ (SWUC-T-THO-05-02), Beibeng Township, Damu, 29°14'51"N, 95°11'1"E, elev. 924 m, 28 June 2018, L.Y. Wang, Y.N. Mu and Z.S. Wu leg. • 1 ♀ (SWUC-T-THO-05-03), Dexing Township, Guoguotang, 29°19.560'N, 95°16.360'E, elev.1025 m, 29 June 2018, L.Y. Wang, Y.N. Mu and Z.S. Wu leg. • 1 ♀ (SWUC-T-THO-05-04), Beibeng Township, Bayang Village, 29°12'49"N, 95°5'43"E, elev. 738 m, 7 July 2023, Z.S. Zhang, L.Y. Wang, Q.L. Lu and X.L. Chen leg. • 1 ♀ (SWUC-T-THO-05-05), Beibeng Township, Badeng Village, 29°16'28"N, 95°10'7"E, elev. 851 m, 7 July 2023, Z.S. Zhang, L.Y. Wang, Q.L. Lu and X.L. Chen leg. • 3 ♀ (SWUC-T-THO-05-06~08), Mirage Observation Deck, 29°20'36"N, 95°20'43"E, elev. 1297 m, 8 July 2023, Z.S. Zhang, L.Y. Wang, Q.L. Lu and X.L. Chen leg.

#### Etymology.

The specific name is derived from the type locality (medog = motuo); it is a noun in apposition.

#### Diagnosis.

The male of this new species resembles *S.comminum* Tang & Li, 2010 (Tang and Li 2010: 70, figs 52A–D, 54A, B) in the bulb of the same shape and the embolus origin in the same position, but it differs from the latter by the absence of an intermedial tibial apophysis (vs presence in *S.comminum*), the conical tutacular apophysis (vs hook-shaped in *S.comminum*) (Fig. 9A, B). The female of this new species can be distinguished from all other *Spilosynema* species in having the epigynal plate with a hood.

#### Description.

**Male** holotype (Fig. 3I) total length 4.20. Prosoma 2.12 long, 1.98 wide; Opisthosoma 2.18 long, 1.65 wide. Carapace yellow brown. Eye sizes and interdistances: AME 0.11, ALE 0.21, PME 0.09, PLE 0.15; AME–AME 0.19, AME–ALE 0.18, PME–PME 0.23, PME–PLE 0.34, ALE–PLE 0.26. MOA 0.45 long, anterior width 0.40, posterior width 0.40. Clypeus height 0.19. Chelicerae brown, with 2 promarginal and 2 retromarginal teeth. Labium and endites brown, longer than wide. Sternum brown and scutellate, with brown hairs. Leg measurements: I 10.49 (2.89, 3.53, 2.64, 1.43); II 10.38 (2.88, 3.59, 2.55, 1.36); III 5.47 (1.72, 1.99, 0.98, 0.78); IV 5.96 (1.99, 2.11, 1.11, 0.75). Leg formula: 1243. Opisthosoma oval, yellow-brown, with a scaly spot. Spinnerets brown.

***Palp*** (Fig. 9A, B). Tibia as long as wide. Ventral tibial apophysis strong, tip slightly hooked. Retrolateral tibial apophysis curved and strongly sclerotized. Cymbium base with two apophyses. Bulb flat. Embolus filiform, originating at approximately 8-o’clock position, curved along with bulb, tip staying in cymbial furrow.

**Female** paratype (SWUC-T-THO-05-02, Fig. 3J) total length 5.23. Prosoma 2.28 long, 2.18 wide; opisthosoma 2.93 long, 2.58 wide. Eye sizes and interdistances: AME 0.11, ALE 0.22, PME 0.10, PLE 0.14; AME–AME 0.25, AME–ALE 0.20, PME–PME 0.30, PME–PLE 0.44, ALE–PLE 0.35. MOA 0.45 long, anterior width 0.49, posterior width 0.53. Clypeus height 0.24. Leg measurements: I 10.10 (2.97, 3.64, 2.28, 1.21); II 9.98 (3.02, 3.49, 2.29, 1.18); III 5.10 (1.55, 1.90, 0.98, 0.67); IV 5.58 (1.82, 1.92, 1.10, 0.74). Leg formula: 1243.

***Epigyne*** (Fig. 9C, D). Epigynal plate wider than long, with a hood at middle. Copulatory openings slit-like, distant from each other. Copulatory ducts S-like. Spermathecae peanut-shaped. Fertilization ducts crescent-shaped.

#### Variation.

Females (*n = 7*) total length 5.23–5.93.

#### Distribution.

China (Xizang, Medog) (Fig. 11).

##### ﻿Genus *Thomisus* Walckenaer, 1805 (蟹蛛属)

### ﻿ Thomisus yarang

Taxon classificationAnimaliaAraneaeThomisidae

Wang, Lu & Zhang sp. nov.

7FE10D02-AF30-5DA1-BB19-1BB68CF2FA31

https://zoobank.org/D3B67A42-9792-4B5D-BB7B-077186A88D48

Figs 3K–L , 10 , 11

#### Type material.

***Holotype*** • ♂ (SWUC-T-THO-06-01), China, Xizang, Medog County, Medog Town, Yarang Village, 29°17.758'N, 95°16.827'E, elev. 761 m, 28 June 2018, L.Y. Wang, Z.S. Wu and Y.N. Mu leg. ***Paratypes***: • 1 ♂ 1 ♀ (SWUC-T-THO-06-02 and SWUC-T-THO-06-02-03), same data as for holotype.

#### Etymology.

The specific name is derived from the type locality; it is a noun in apposition.

#### Diagnosis.

**Figure 10.**
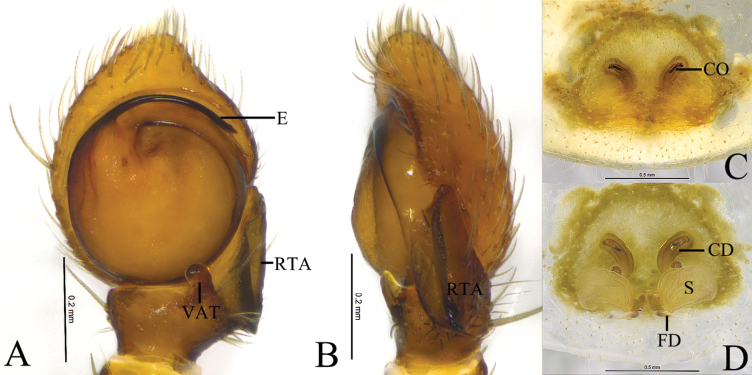
*Thomisusyarang* Wang, Lu & Zhang, sp. nov. **A, B** holotype male **C, D** paratype female **A** male left palp, ventral view **B** same, retrolateral view **C** epigyne, ventral view **D** same, dorsal view. Abbreviations: CD = copulatory duct; CO = copulatory opening; E = embolus; FD = fertilization duct; RTA = retrolateral tibial apophysis; VTA = ventral tibial apophysis; S = spermathecal.

The new species resembles *T.labefactus* Karsch, 1881 (Song and Zhu 1997: 167, fig. 117A–E) in having a similar long and curved embolus, but it differs from the latter by the long, digitiform ventral tibial apophysis (vs short and conical in *T.labefactus*), the groove-shaped retrolateral tibial apophysis (vs base stretches in *T.labefactus*), and the arc-shaped copulatory ducts (vs semicircular in *T.labefactus*) (Fig. 10).

#### Description.

**Male** holotype (SWUC-T-THO-06-01, Fig. 3K) total length 3.84. Prosoma 1.90 long, 1.98 wide; opisthosoma 2.24 long, 2.31 wide. Carapace brown, with a great many small denticles. Eye sizes and interdistances: AME 0.12, ALE 0.14, PME 0.11, PLE 0.10; AME–AME 0.27, AME–ALE 0.32, PME–PME 0.57, PME–PLE 0.42, ALE–PLE 0.24. MOA 0.39 long, anterior width 0.51, posterior width 0.75. Clypeus height 0.30. Chelicerae brown, with 3 promarginal and 2 retromarginal teeth. Leg measurements: I 5.35 (1.75, 1.87, 0.97, 0.76); II 5.48 (1.82, 1.93, 0.95, 0.78); III 2.94 (0.95, 1.06, 0.49, 0.44); IV 3.11 (1.04, 1.02, 0.58, 0.47). Leg formula: 2143. Opisthosoma brown, dorsum with small spines, venter yellow.

***Palp*** (Fig. 10A, B). Tibia as long as wide. Ventral tibial apophysis blunt, digitiform. Retrolateral tibial apophysis large, wide, groove-shaped. Embolus originating at approximately 11-o’clock position, curved along with bulb.

**Female** paratype (SWUC-T-THO-06-03, Fig. 3L) total length 10.73. Prosoma 5.42 long, 5.19 wide; opisthosoma 5.84 long, 8.29 wide. Carapace yellowish. Eye sizes and interdistances: AME 0.20, ALE 0.23, PME 0.16, PLE 0.18; AME–AME 0.56, AME–ALE 0.71, PME–PME 1.09, PME–PLE 0.73, ALE–PLE 0.37. MOA 0.79 long, anterior width 0.95, posterior width 1.43. Clypeus height 0.65. Leg measurements: I 15.60 (4.99, 5.75, 2.81, 2.05); II 14.69 (4.74, 5.51, 2.69, 1.75); III 8.68 (2.92, 3.14, 1.62, 1.00); IV 10.14 (3.30, 3.72, 1.93, 1.19). Leg formula: 2143. Opisthosoma white, with a brown spot.

***Epigyne*** (Fig. 10C, D). Epigynal plate oval. Copulatory openings arc-like, distant from each other. Copulatory ducts arc-like, three times longer than wide. Spermathecae transparent, spherical. Fertilization ducts crescent-shaped.

#### Variation.

Males (*n = 2*) total length 3.29–3.84.

#### Distribution.

**Figure 11.**
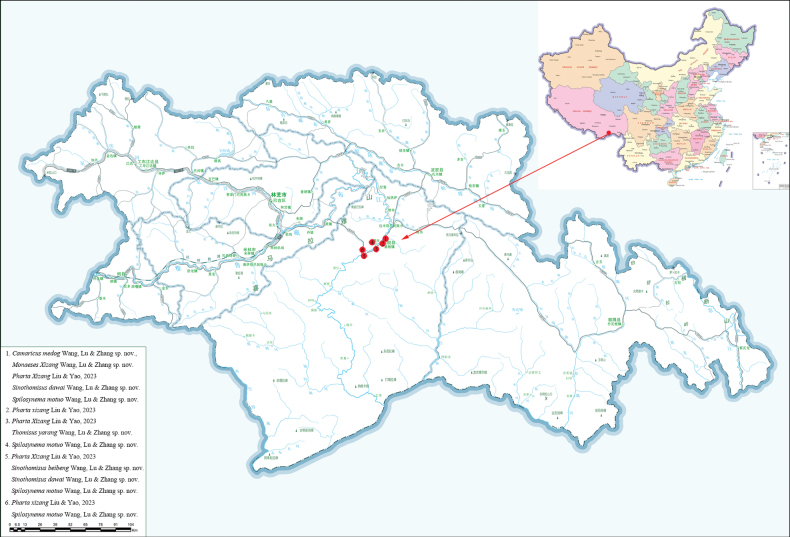
Distribution of crab spiders from Medog, Xizang, China.

Known only from the type locality, Medog, Xizang, China (Fig. 11).

## ﻿Discussion

Among these seven species of crab spiders described here, only *Phartaxizang* Liu & Yao, 2023 was active at night. The rest of the species were collected during the day. All six new species were collected from flower beds along the side of a road, except *Monaesesxizang* Wang, Lu & Zhang, sp. nov. which was also collected from a road guardrail. It may be due to the abundance of precipitation, which makes the forest too dense and humid, so the crab-spider spiders go out of the forest to live at the edges.

## Supplementary Material

XML Treatment for Camaricus medog

XML Treatment for Monaeses xizang

XML Treatment for Pharta xizang

XML Treatment for Sinothomisus beibeng

XML Treatment for Sinothomisus dawai

XML Treatment for Spilosynema motuo

XML Treatment for Thomisus yarang

## ﻿Additional information

### Author ORCIDs

Lu-Yu Wang https://orcid.org/0000-0002-5250-3473

Yan-Nan Mu https://orcid.org/0000-0002-2504-673X

Qian-Le Lu https://orcid.org/0000-0001-6291-9117

Yong-Qiang Xu https://orcid.org/0000-0002-1889-9531

Feng Zhang https://orcid.org/0000-0002-3347-1031

Zhi-Sheng Zhang https://orcid.org/0000-0002-9304-1789

### Data availability

All of the data that support the findings of this study are available in the main text.

## References

[B1] Álvarez-Padilla F Hormiga G (2007) A protocol for digesting internal soft tissues and mounting spiders for scanning electron microscopy. The Journal of Arachnology 35(3): 538–542. 10.1636/Sh06-55.1

[B2] Benjamin SP (2011) Phylogenetics and comparative morphology of crab spiders (Araneae: Dionycha, Thomisidae). Zootaxa 3080: 1–108. 10.11646/zootaxa.3080.1.1

[B3] Chang WJ Li SQ (2020) Thirty-one new species of the spider genus *Leclercera* from Southeast Asia (Araneae, Psilodercidae). ZooKeys 913: 1–87. 10.3897/zookeys.913.48650 32132849 PMC7044255

[B4] Chang WJ Yao ZY Li SQ (2020) Twenty-eight new species of the spider genus *Merizocera* Fage, 1912 (Araneae, Psilodercidae) from South and Southeast Asia. ZooKeys 961: 41–118. 10.3897/zookeys.961.53058 32904093 PMC7449989

[B5] Cheng WH Bian DJ Tong YF Li SQ (2021) A new genus and two new species of oonopid spiders from Tibet, China (Araneae, Oonopidae). ZooKeys 1052: 55–69. 10.3897/zookeys.1052.66402 34393552 PMC8346458

[B6] Chu C Lu Y Li SQ Yao ZY (2022) Taxonomic notes on eleven species of the subfamily Cteninae (Araneae, Ctenidae) from Asia. Biodiversity Data Journal 10: e96003. 10.3897/BDJ.10.e96003 PMC9836443 36761640

[B7] Fu YN Zhu MS (2008) A new species of the genus *Pseudopoda* from China (Araneae, Sparassidae). Acta Zootaxonomica Sinica 33: 657–659.

[B8] Jiang TY Zhao QY Li SQ (2018) Sixteen new species of the genus *Pseudopoda* Jäger, 2000 from China, Myanmar, and Thailand (Sparassidae, Heteropodinae). ZooKeys 791: 107–161. 10.3897/zookeys.791.28137 PMC6205991 30386156

[B9] Li CZ Yao YB Xiao YH Liu KK (2023) Two new thomisid species (Arachnida, Araneae, Thomisidae) from China and Vietnam, with the first descriptions of the males of *Borboropactuslongidens* Tang & Li, 2010 and *Stephanopisxiangzhouica* Liu, 2022. ZooKeys 1159: 169–187. 10.3897/zookeys.1159.102601 37213528 PMC10193142

[B10] Li R Wang GL Zhang YH Guo ZH (2024) Study on precipitation characteristics in Medog, southeastern Tibetan Plateau. Meteorololgical Monthly 50(3): 303–317. 10.7519/j.issn.1000-0526.2023.111801

[B11] Lin YJ Yan XY Li SQ (2022) Two new species of the genus *Chilobrachys* (Araneae, Theraphosidae) from China. ZooKeys 1081: 99–109. 10.3897/zookeys.1081.77072 35087297 PMC8776719

[B12] Lu JZ Wang LY Zhang ZS (2023) A new species of *Hygropoda* from Tibet, China (Agelenidae: Pisauridae). Acta Arachnologica Sinica 32(2): 122–126. 10.3969/j.issn.1005-9628.2023.02.011

[B13] Mi XQ Zhang T Wang C (2024) Description of two species of the orb-weaver spider genus *Argiope* Audouin, 1826 (Araneae, Araneidae) from Xizang, China. Biodiversity Data Journal 12(e125601): 1–14. 10.3897/BDJ.12.e125601 PMC11250172 39015799

[B14] Song DX Zhu MS (1997) Fauna Sinica: Arachnida: Araneae: Thomisidae, Philodromidae. Science Press, Beijing, 259 pp.

[B15] Tang G Li SQ (2010) Crab spiders from Xishuangbanna, Yunnan Province, China (Araneae, Thomisidae). Zootaxa 2703: 1–105. 10.11646/zootaxa.2703.1.1

[B16] Tang G Yin CM Griswold C Peng XJ (2006) Description of *Sinothomisus* gen. nov. with a new species from Yunnan Province, China (Araneae, Thomisidae). Zootaxa 1366: 61–68. 10.11646/zootaxa.1366.1.4

[B17] Tong YF Bian DJ Li SQ (2023) Three new species of the genus *Ischnothyreus* Simon, 1893 and the discovery of the male of I. linzhiensis Hu, 2001 from Tibet, China (Araneae, Oonopidae). ZooKeys 1152: 119–131. 10.3897/zookeys.1152.100341 37214739 PMC10193446

[B18] Wang Z Peng JS (2022) The rich treasures of the primeval forest in Medog. Forest & Humankind 7: 62–75.

[B19] Wang LY Zhang ZS (2020) The first record of *Zantheres* and *Z.gracillimus* Thorell, 1887 (Araneae, Lycosidae) from China. Acta Arachnologica Sinica 29(2): 99–102. 10.3969/j.issn.1005-9628.2020.02.004

[B20] Wang C Mi XQ Peng XJ (2016) A new species of *Pharta* Thorell, 1891 (Araneae: Thomisidae) from China. Oriental Insects 50(3): 129–134. 10.1080/00305316.2016.1197163

[B21] Wang LY Peng XJ Zhang ZS (2021) *Serratacosa*, a new genus of Lycosidae (Araneae) from the southern slopes of the Eastern Himalayas. European Journal of Taxonomy 762: 96–107. 10.5852/ejt.2021.762.1455

[B22] Wang C Mi XQ Li SQ (2024) Eleven species of jumping spiders from Sichuan, Xizang, and Yunnan, China (Araneae, Salticidae). ZooKeys 1192: 141–178. 10.3897/zookeys.1192.114589 38425441 PMC10902788

[B23] Wang LY Irfan M Zhang F Zhang Z.S (2024a) On three species of ctenids (Araneae: Ctenidae) from Tibet, China. Zootaxa 5458(1): 119–129. 10.11646/zootaxa.5458.1.7 39646946

[B24] Wang LY Mu YN Lu F Xu YQ Zhang ZS (2024b) Ant-eating spiders from Xizang, China (Araneae, Zodariidae). ZooKeys 1200: 183–198. 10.3897/zookeys.1200.120528 38756346 PMC11096724

[B25] Xiong F Liu ZP Zhang ZS (2017) Review on the jumping spider genus Hyllus from China (AraneaeSalticidae). Acta Arachnologica Sinica 26(1): 22–26. 10.3969/j.issn.1005-9628.2017.01.005

[B26] Yang ZY Zhang JX (2024) On eight species of Chrysillini from Xizang, China (Araneae: Salticidae: Salticinae). Zootaxa 5447(2): 151–187. 10.11646/zootaxa.5447.2.1 39645839

[B27] Yin CM Wang JF Xie LP Peng XJ (1990) New and newly recorded species of the spiders of family Araneidae from China (Arachnida, Araneae). In: Spiders in China: One Hundred New and Newly Recorded Species of the Families Araneidae and Agelenidae. Hunan Normal University Press, Changsha, 1–171.

[B28] Zhang QQ Lin YC (2018) A review of the spider genus Sinanapis, with the description of a new species from Tibet (Araneae, Anapidae). ZooKeys 790: 45–61. 10.3897/zookeys.790.25793 PMC6198031 30364855

[B29] Zhang F Yu K (2021) Description of two species of *Conothele* Thorell, 1878 from Tibet, China (Mygalomorphae: Halonoproctidae). Journal of Hebei University, Natural Science Edition 41(5): 581–586.

[B30] Zhang L Zhang F (2023) First report of the genus *Apochinomma* Pavesi, 1881 from China, with description of a new species (Araneae, Corinnidae, Castianeirinae). Zootaxa 5323(3): 446–450. 10.11646/zootaxa.5323.3.11 38220952

[B31] Zhang F Zhu MS Song DX (2006) A review of pholcid spiders from Tibet, China (Araneae, Pholcidae). Journal of Arachnology 34(1): 194–205. 10.1636/H04-22.1

[B32] Zhang F Zhu MS Song DX (2007) Two new species of the genus *Clubiona* from Xizang autonomous region, China (Araneae, Clubionidae). Journal of the Liaoning Normal University, Natural Science Edition 30: 90–92.

[B33] Zhang H Zhu Y Zhong Y Jäger P Liu J (2023) A taxonomic revision of the spider genus *Pseudopoda* Jäger, 2000 (Araneae: Sparassidae) from East, South and Southeast Asia. Megataxa 9(1): 1–304. 10.11646/megataxa.9.1.1

[B34] Zhu MS Wang XP Zhang ZS (2017) Fauna Sinica: Invertebrata Vol. 59: Arachnida: Araneae: Agelenidae and Amaurobiidae. Science Press, Beijing, 727 pp.

[B35] Zhu WH Yao ZY Zheng G Li SQ (2020) The *Belisana* spiders (Araneae: Pholcidae) from Tibet, China. Zootaxa 4802(1): 111–128. 10.11646/zootaxa.4802.1.7 33056635

